# The clinical significance of 5% change in vital capacity in patients with idiopathic pulmonary fibrosis: extended analysis of the pirfenidone trial

**DOI:** 10.1186/1465-9921-12-93

**Published:** 2011-07-15

**Authors:** Hiroyuki Taniguchi, Yasuhiro Kondoh, Masahito Ebina, Arata Azuma, Takashi Ogura, Yoshio Taguchi, Moritaka Suga, Hiroki Takahashi, Koichiro Nakata, Atsuhiko Sato, Yukihiko Sugiyama, Shoji Kudoh, Toshihiro Nukiwa

**Affiliations:** 1 Dept of Respiratory Medicine and Allergy, Tosei General Hospital, Seto, Aichi, Japan; 2 Dept of Respiratory Medicine, Tohoku University Graduate School of Medicine, sendai, Japan; 3 Dept of Internal Medicine, Nippon Medical School, Tokyo, Japan; 4 Dept of Respiratory Medicine, Kanagawa Cardiovascular and Respiratory Center, Yokohama, Japan; 5 Dept of Respiratory Medicine, Tenri Hospital, Tenri, Japan; 6 Dept of respiratory medicine, Saiseikai Kumamoto Hospital, Kumamoto, Japan; 7 Third Dept of Internal Medicine, Sapporo Medical University Hospital, Sapporo, Japan; 8 Dept of respiratory Medicine, Nakata Clinic, Tokyo, Japan; 9 Dept of respiratory Medicine, Kyoto Preventive Medical Center, Kyoto, Japan; 10 Dept of Medicine, Division of Pulmonary Medicine, Jichi Medical University, Tochigi, Japan

## Abstract

**Background:**

Our phase III clinical trial of pirfenidone for patients with idiopathic pulmonary fibrosis (IPF) revealed the efficacy in reducing the decline of vital capacity (VC) and increasing the progression-free survival (PFS) time by pirfenidone. Recently, marginal decline in forced VC (FVC) has been reported to be associated with poor outcome in IPF. We sought to evaluate the efficacy of pirfenidone from the aspects of 5% change in VC.

**Methods:**

Improvement ratings based on 5% change in absolute VC, i.e., "improved (VC ≥ 5% increase)", "stable (VC < 5% change)", and "worsened (VC ≥ 5% decrease)" at month 3, 6, 9 and 12 were compared between high-dose pirfenidone (1800 mg/day; n = 108) and placebo (n = 104) groups, and (high-dose and low-dose (1200 mg/day; n = 55)) pirfenidone (n = 163) and placebo groups. PFS times with defining the disease progression as death or a ≥ 5% decline in VC were also compared between high-dose pirfenidone and placebo groups, and low-dose pirfenidone and placebo groups. Furthermore, considering "worsened" and "non-worsened (improved and stable)" of the ratings at months 3 and 12 as "positive" and "negative", respectively, and the positive and negative predictive values of the ratings were calculated in each group.

**Results:**

In the comparison of the improvement ratings, the statistically significant differences were clearly revealed at months 3, 6, 9, and 12 between pirfenidone and placebo groups. Risk reductions by pirfenidone to placebo were approximately 35% over the study period. In the comparison of the PFS times, statistically significant difference was also observed between pirfenidone and placebo groups. The positive/negative predictive values in placebo and pirfenidone groups were 86.1%/50.8% and 87.1%/71.7%, respectively. Further, the baseline characteristics of patients worsened at month 3 had generally severe impairment, and their clinical outcomes including mortality were also significantly worsened after 1 year.

**Conclusions:**

The efficacy of pirfenidone in Japanese phase III trial was supported by the rating of 5% decline in VC, and the VC changes at month 3 may be used as a prognostic factor of IPF.

**Trial Registration:**

This clinical trial was registered with the Japan Pharmaceutical Information Center (JAPIC) on September 13^th^, 2005 (Registration Number: JAPICCTI-050121).

## Background

Idiopathic pulmonary fibrosis (IPF) is a chronic, progressive, and fatal lung disease for which there is no known cause or proven effective therapy [1,2]. Pirfenidone (5-methyl-1-phenyl-2-[1H]-pyridone; Shionogi & Co., Ltd., Osaka, Japan; MARNAC Inc., Dallas, TX, USA) [3-6] is a pyridone compound with therapeutic potential for IPF that has been shown in animal models to have wide-ranging effects including antifibrotic, anti-inflammatory and antioxidant activity, although its precise mode of action is unknown [2,7-11]. A multi-centere, double-blind, placebo-controlled, randomized phase III clinical trial was conducted in Japanese patients with IPF to determine the efficacy and safety of pirfenidone over 52 weeks [12]. Significant differences were observed in the decline of vital capacity (VC; primary endpoint) between placebo group and high-dose (1800 mg/day) group; and in the secondary end point, the progression free survival (PFS) time, between the two groups. Treatment with pirfenidone was associated with a decreased rate of decline in VC and increased the PFS time over 52 weeks.

A 10% change in forced VC (FVC) have been reported to be a promising prognostic indicator, because patients with ≥ 10% decline in FVC within 6 or 12 months have a poor prognosis [13-15]. In the treatment guidelines published by the American Thoracic Society (ATS)/European Respiratory Society (ERS) as well, a ≥ 10% change in FVC and ≥ 15% change in diffusing capacity of the lung for carbon monoxide (DLCO) are described as indices of improvement or worsening of disease [16]. To evaluate changes over a period from 6 months to 1 year, however, the method using a 10% change in FVC as an index is not sensitive enough and may not be suitable for actual clinical setting. Recently, Zappala *et al. *have reported that marginal decline in FVC is associated with a poor outcome in IPF [17]. In this report, the authors demonstrated that IPF patients had a significantly poor prognosis when the decline in FVC after 6 months was either 5% to 10% or ≥ 10%. This information is considered useful for selecting patients with progressive disease and evaluating therapeutic effects in clinical studies.

Based on this report, we reviewed the efficacy of pirfenidone in the phase III trial in an exploratory manner using a 5% change in VC as indices, evaluated the coincidence of the ratings based on 5% change in VC between months 3 and 12, and examined the usefulness and significance of the 5% change.

## Methods

### Overall Study Design

This study was a multicentre, double-blind, randomized, placebo-controlled trial. The diagnosis of IPF was in accordance with the ATS/ERS Consensus statement [16] and 4^th ^version of the guideline of clinical diagnostic criteria for idiopathic interstitial pneumonia in Japan [18]. Eligible patients were adults (20 to 75 years old) with IPF diagnosis based on above criteria and meeting the following SpO_2 _criteria: 1) demonstrate oxygen desaturation of > 5% difference between resting SpO_2 _and the lowest SpO_2 _during a 6-minute steady-state exercise test (6MET), and 2) the lowest SpO_2 _during the 6MET > 85% while breathing air. Using the data in our pirfenidone phase III trial [12], we performed a series of exploratory analyses of physiologic variables and characteristics in patients receiving high-dose pirfenidone [1800 mg/day], low-dose pirfenidone [1200 mg/day] or placebo.

### Setting, Participants, and Randomization

In this phase III study, 325 patients were screened at 73 centers in Japan, and 275 patients were randomized to one of the three groups: the high-dose, low-dose and placebo groups. Of the 275 patients, 267 (108, 55 and 104 patients in the high-dose, low-dose and placebo groups, respectively) were deemed eligible for the full analysis set (FAS). Eight patients were excluded due to having no post-baseline data.

### Measurements

The primary endpoint was the change in VC from baseline to Week 52. Secondary endpoints were PFS time and the change in the lowest SpO_2 _during 6MET. VC was measured every 4 weeks, while the lowest SpO_2 _during the 6MET and other PFTs were determined every 12 weeks.

### Statistical Analysis

In order to examine the characteristics of the improvement ratings and PFS based on 5% change in VC in the comparison of efficacy among treatment groups, and the clinical significance of the 5% decline in VC at month 3, we performed following analyses. Significance level of tests was set at 0.1 (two-sided) according to the phase III study [12].

#### • Categorical analysis based on 5% change in VC

Improvement ratings were defined based on 5% relative changes in absolute VC from baseline as "improved (≥ 5% increase)", "stable (< 5% change)", and "worsened (VC ≥ 5% decrease)", using VC values measured at 12, 28, 40, and 52 weeks after the start of treatment, and these ratings were used as those at months 3, 6, 9, and 12, respectively. Then, the distributions of the improvement ratings were compared between, high-dose pirfenidone (n = 108) and placebo (n = 104) groups, and (high- and low-dose) pirfenidone (n = 163) and placebo (n = 104) groups, with Wilcoxon rank sum test. The risk ratio was also calculated as the ratio of proportion of "worsened" in pirfenidone group to the proportion in placebo group at each time point. The principle of the last observation carried forward (LOCF) was adopted to impute missing values if patient data were available for ≥ 4 weeks after the baseline. The number of patients prematurely dropped and for whom missing observations were imputed was shown in online supplemental materials of the preceding reports in details [12,19].

#### • Comparison of PFS times based on 5% decline in VC or death

PFS times by definition of disease progression as death or ≥ 5% relative decline in absolute VC were obtained. (In our previous paper, we used ≥ 10% instead of ≥ 5% decline in VC to define PFS times [12].) Then, the cumulative PFS rates were estimated with Kaplan-Meier (K-M) method and the distributions of PFS times were compared with log-rank test between high-dose pirfenidone and placebo groups, and low-dose pirfenidone and placebo groups. In addition, the disease progression was defined also by ≥ 5% decline in VC on two consecutive data points or death and similar analyses of PFS times thus defined were performed.

#### • Coincidence of the improvement ratings based on 5% change in VC at months 3 and 12, in terms of positive and negative predictive values

In order to examine the coincidence of the improvement ratings at month 3 and 12, that were derived as shown in the subsection "Categorical analysis based on 5% change in VC", we calculated positive and negative predictive values in high- and low-dose pirfenidone and placebo groups, and compared the positive and negative predictive values between the 2 (or pirfenidone and placebo) groups. Then, "worsened" and "non-worsened (stable or improved)" were considered "positive" and "negative", respectively.

#### • Comparison of the baseline characteristics between 'worsened' and 'non-worsened' patients at month 3

To examine the profiles of patients with ≥ 5% and < 5% decline in VC ("worsened" and "non-worsened" patients) at month 3, the baseline characteristics (i.e. age, body mass index (BMI), alveolar-arterial oxygen tension (PaO_2_), SpO_2_, VC, %VC, total lung capacity (TLC), %TLC, DLCO, %DLCO, KL-6, surfactant protein (SP)-A, SP-D, and dyspnea in daily living assessed with Hugh-Jones (H-J) classification [20]) between "worsened" and "non-worsened" patients at month 3 were compared with Welch's t-test.

#### • Comparison of the clinical outcome after 1 year between 'worsened' and 'non-worsened' patients at month 3

The clinical outcome (i.e. H-J classification, death, and acute exacerbation) after 1 year were compared between "worsened" and "non-worsened" patients at month 3. Analysis of the H-J classification was performed with Welch's T-test. Analyses of the mortality ratio and incidence of acute exacerbation were with Fisher's exact test.

#### • Comparison of PFS times with origin at month 3 between 'worsened' and 'non-worsened' patients at month 3

PFS times with origin at month 3 were obtained in a similar manner as described above. Then, the cumulative PFS rates were estimated with K-M method and the distributions of PFS times were compared with log-rank test between "worsened" and "non-worsened" patients at month 3.

## Results

### Categorical analysis based on 5% change in VC

**Figure 1:**
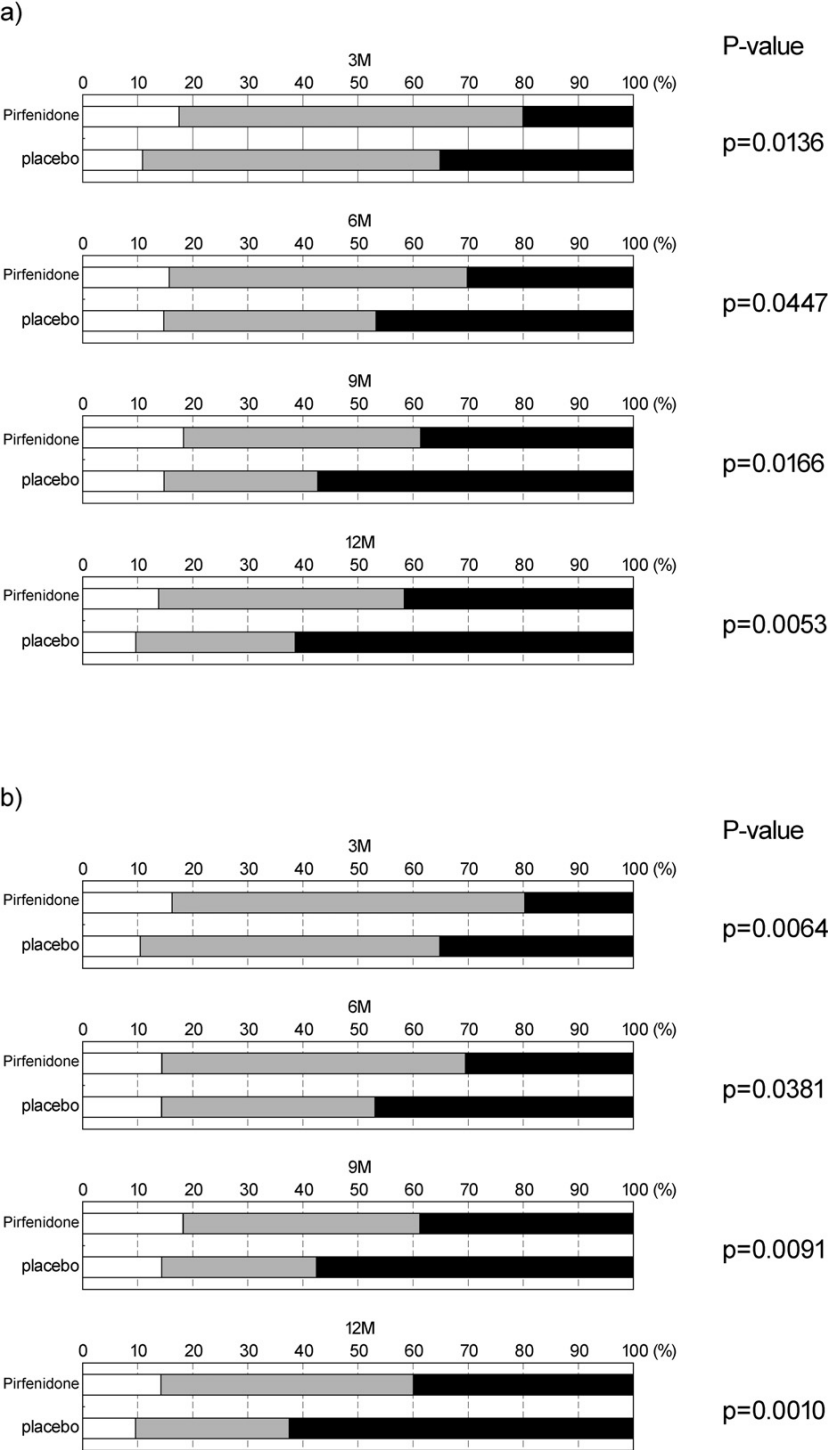
**Categorical analysis based on 5% changes in VC at months 3, 6, 9, and 12**. Improvement ratings based on 5% changes in VC were defined as "improved (VC 5% increase)", "stable (VC < 5% change)", and "worsened (VC 5% decrease)", using VC values measured at months 3, 6, 9, and 12. a) high-dose vs. placebo groups, b) pirfenidone-treated (high + low-dose) vs. placebo groups. The results are shown by the frequencies of improved (white areas), stable (gray areas), and deteriorated (black areas). P-values by Wilcoxon's test are indicated at the right.

Improvement ratings (improved, stable, worsened) based on 5% relative change in absolute VC at months 3, 6, 9 and 12 are shown in Figures 1-a (for high-dose pirfenidone and placebo groups) and 1-b (for high- and low-dose pirfenidone and placebo groups). Significant differences in the distributions of the ratings were consistently observed between high-dose pirfenidone and placebo groups (p = 0.0136, 0.0447, 0.0166, and 0.0053, Risk ratio; 0.578, 0.640, 0.671, and 0.665 at months 3, 6, 9, and 12, respectively) (Figure 1-a). Significant differences were also seen between high- and low- dose pirfenidone and placebo groups (p = 0.0064, 0.0381, 0.0091, and 0.0010, Risk ratio; 0.561, 0.652, 0.674, and 0.642 at months 3, 6, 9, and 12, respectively) (Figure 1-b), and between low-dose pirfenidone and placebo groups (**data not shown**). At months 6, 9, and 12, the risk ratios in (high- and low-dose) pirfenidone group to those in placebo group were approximately 65%., and the risks to be judged 'worsened' were consistently lower in pirfenidone group by approximately 35%.

### Evaluation using modified progression-free survival based on 5% decline in VC or death

**Figure 2:**
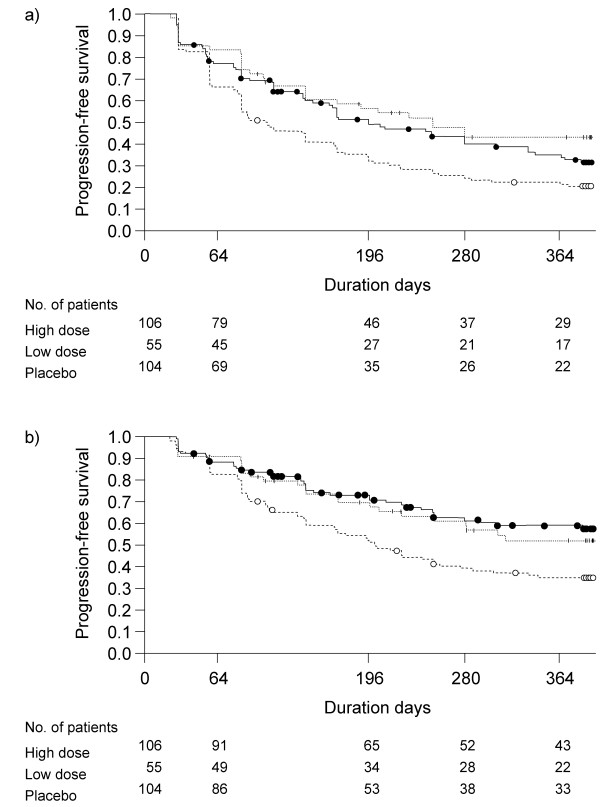
**Kaplan-Meier plot of Progression-Free Survival (PFS) times in groups of IPF patients**. a) The disease progression was defined by a ≥ 5% decline in VC from baseline or death. b) The disease progression was defined by a ≥ 5% decline in VC from baseline on two consecutive occasions or death. Solid line: high-dose; broken line: low-dose; bold broken line: placebo. The distribution of PFS times were compared with log-rank test.

The modified progression of disease was defined by a ≥ 5% decline in absolute VC from baseline or death. K-M plots of PFS times based on the definition and the results of comparison of the distributions of PFS times among the groups with log-rank test are shown in Figure 2-a. Significant differences were shown in the distributions of PFS times between high-dose and placebo groups (p = 0.0149), and between low-dose and placebo groups (p = 0.0034) (Figure 2-a ), and between (high-dose and low-dose) pirfenidone and placebo groups (p = 0.0015) (**data not shown**).

The progression of disease was also defined by ≥ 5% decline in VC on two consecutive data points or death, and K-M plots of the PFS times thus defined and the results of comparison with log-rank test are shown in Figure 2-b. Significant differences in the PFS times were seen between high-dose and placebo groups (p = 0.0011), between low-dose and placebo groups (p = 0.0349) (Figure 2-b ), and between (high- and low-dose) pirfenidone and placebo groups (p = 0.0006) **(data not shown)**.

### Positive predictive value, negative predictive value with the ratings at month 3

Positive and negative predictive values with the ratings at month 3 in the prediction of those at month 12 in placebo and pirfendone (high- + low-dose) groups are shown in Table 1. In the placebo group, a ≥ 5% decline in VC at month 3 was still present at month 12 at highly rate (positive predictive value; 86.1% (31/36)) and no decline at month 3 was stable at month 12 at a rate of about 50% (negative predictive value; 50.8% (34/67)). On the other hand, in the treated (high- and low-dose pirfenidone) groups, decline at month 3 was still highly present (positive predictive value; 87.1% (27/31), nearly equal to one in the placebo group), and no decline at month 3 was also still stable at month 12 in relatively highly rate (negative predictive value; 71.7% (91/127) (Table 1 ). To put it briefly, the positive predictive values for pirfenidone and placebo groups were 87.1% and 86.1% respectively, and the difference was not significant. On the other hand, the negative predictive values for pirfenidone and placebo groups were 71.7% and 50.8%, respectively, and significant difference was seen (p = 0.0046).

**Table 1:** Positive and negative predictive values of the ratings at month 3 in the prediction of the ratings at month 12

**Placebo group(n = 103)**				
		**12M worsened/non-worsened**		
		
		**Worsened**	**Non-worsened**	
		
	Worsened	31 (86.1%)	5 (13.9%)	36
3M worsened/non-worsened				
	Non-worsened	33 (49.2%)	34 (50.8%)	67
		
		64	39	103
				
**Pirfenidone(high + low-dose) group (n = 158)**				
		**12M worsened/non-worsened**		
		
		**Worsened**	**Non-worsened**	
		
	Worsened	27 (87.1%)	4 (12.9%)	31
3M worsened/non-worsened				
	Non-worsened	36 (28.4%)	91 (71.7%)	127
		
		63	95	158

### Comparison of the baseline characteristics between 'worsened' and 'non-worsened' patients at month 3

The baseline characteristics between 'worsened' and 'non-worsened' patients at month 3 were compared. Patients with VC declined by 5% at month 3 generally had lower means of BMI, PaO_2_, VC, %VC, TLC, %TLC, and DLCO at baseline (p = 0.0011, 0.0047, 0.0036, 0.0127, 0.0219, 0.0722, 0.0639, respectively), and had higher means of SP-A, SP-D and H-J classification score at baseline (p = 0.0281, 0.0344, 0.0765, respectively) (Table 2).

**Table 2:** Summary statistics of baseline characteristics for patients with ≥ 5% and < 5% decline in VC at month 3

Characteristics		5% decline in VC at Month 3			
		No	Yes	Total*	P-value
Age	Subjects	194	67	261	0.3623
	Mean ± S.D.	65.1 ± 6.5	64.1 ± 7.9	64.9 ± 6.9	

BMI	Subjects	194	67	261	0.0011
	Mean ± S.D.	24.7 ± 2.9	23.3 ± 2.9	24.3 ± 3.0	

PaO_2_	Subjects	192	67	259	0.0047
	Mean ± S.D.	81.5 ± 9.6	78.1 ± 7.9	80.6 ± 9.3	

SpO_2_	Subjects	193	67	260	0.1114
	Mean ± S.D.	89.1 ± 2.2	88.6 ± 2.2	89.0 ± 2.2	

VC	Subjects	194	67	261	0.0036
	Mean ± S.D.	2.51 ± 0.67	2.24 ± 0.63	2.44 ± 0.67	

%VC	Subjects	194	67	261	0.0127
	Mean ± S.D.	79.4 ± 17.2	73.3 ± 17.1	77.8 ± 17.3	

TLC	Subjects	193	67	260	0.0219
	Mean ± S.D.	3.76 ± 0.92	3.43 ± 1.01	3.68 ± 0.95	

%TLC	Subjects	193	67	260	0.0722
	Mean ± S.D.	75.0 ± 15.1	70.6 ± 17.8	73.9 ± 15.9	

DLCO	Subject	193	67	260	0.0639
	Mean ± S.D.	9.82 ± 3.23	9.00 ± 3.07	9.61 ± 3.20	

%DLCO	Subjects	193	67	260	0.1768
	Mean ± S.D.	54.4 ± 17.8	51.0 ± 18.0	53.6 ± 17.9	

KL-6	Subjects	194	67	261	0.4436
	Mean ± S.D.	1308.2 ± 771.0	1401.9 ± 889.2	1332.2 ± 802.3	

SP-A	Subjects	194	67	261	0.0281
	Mean ± S.D.	88.0 ± 43.0	108.3 ± 69.7	93.2 ± 51.8	

SP-D	Subjects	194	67	261	0.0344
	Mean ± S.D.	223.1 ± 130.5	282.1 ± 210.9	238.2 ± 156.8	

H-J classification	Subjects	194	67	261	0.0765
	Mean ± S.D.	2.0 ± 0.7	2.2 ± 0.7	2.1 ± 0.7	

* Patients for whom the changes in VC at month 3 couldn't be calculated were deleted from the analysis. The differences in the number of subjects among the variables at column 'Total' were due to missing values at baseline.

TLC, total lung capacity; PaO_2_, arterial oxygen tension; SpO_2_, oxygen saturation by pulse oximetry; DLCO, diffusing capacity for carbon monoxide; SP-A (or D), Surfactant protein-A (or D); BMI, Body Mass Index.

### Comparison of the clinical outcome after 1 year between 'worsened' and 'non-worsened' patients at month 3

We compared the change in H-J classification score from baseline to month 12 with t-test between 2 classes of patients, i.e., those with "worsened (VC ≥ 5% decrease)" and others with "non-worsened (VC < 5% decrease)" at month 3. As a result, significant difference was seen for H-J classification score (p = 0.0002) (Table 3). Additionally, mortality rates for the patients with "non-worsened" and those with "worsened" at month 3 were 2.0% (4/194) and 9.0% (6/67), respectively, and significant difference was recognized (p = 0.0203). Marginal trend was also seen in the prevalence of acute exacerbation between the 2 classes of patients (p = 0.1031) (Table 4).

**Table 3:** Outcome of patients; Change from baseline to month 12 in H-J classification for patients with ≥ 5% and < 5% decline in VC

	5% decline in VC at month 3			
	No	Yes	Total*	P-value
Subjects	194	67	261	
Mean ± S.D.	0.1 ± 0.7	0.6 ± 0.9	0.2 ± 0.8	0.0002

**Table 4:** Outcome after month 12; Mortality ratio and incidence of acute exacerbation in patients with ≥ 5% and < 5% decline in VC

	5% decline in VC at Month 3			
			
	No	Yes	Total*	P-value
Subjects	194	67	261*	

Mortality (%)	4 (2.04)	6 (8.96)	10	0.0203
Acute exacerbation (%)	7 (3.61)	6 (8.96)	13	0.1031

* Patients for whom the changes in VC at month 3 couldn't be calculated were deleted from the analysis.

### Comparison of PFS times with origin at month 3 between 'worsened' and 'non-worsened' patients at month 3

**Figure 3:**
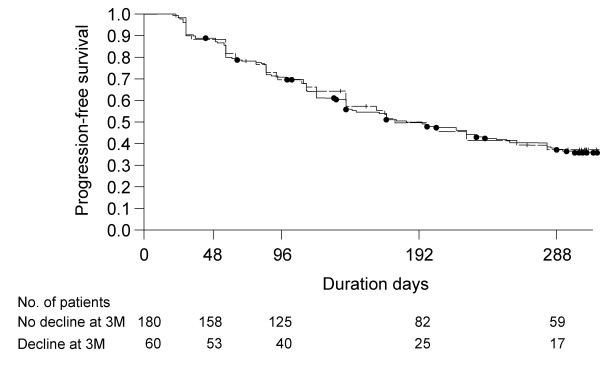
**K-M plot of PFS times with origin at month 3 in groups of patients with and without 5% decline in VC at month 3**. Solid line with closed circle: No decline in VC at month 3; broken line with plus: a ≥ decline in VC at month 3. P-value was 0.8835 with log-rank test.

K-M plot of the PFS times with origin at month 3 for patients with and without 5% decline of VC at month 3, added the result of log-rank test, is shown in Figure 3. There was no significant difference in the distributions of PFS times between the 2 classes of patients (p = 0.8835).

## Discussion

We report that the efficacy of pirfenidone in Japanese phase III trial was supported by the evaluation using the improvement ratings, PFS times and positive/negative predictive values based on 5% decline in VC. Further, the baseline characteristics of patients with ≥ 5% decline at month 3 were generally severe, and the clinical outcomes of those patients including mortality were also significantly worsened after 1 year.

According to a preceding report [12], comparison of the distributions of the improvement ratings (improved, stable, or worsened) based on 10% change in VC did not show significant differences between pirfenidone and placebo groups. The comparison of the ratings using 5% change in VC, however, revealed significant differences between pirfenidone and placebo groups at months 3, 6, 9 and 12 (Figure 1), and approximately 35% reduction in risk in this malignant disease would support the use of pirfenidone in clinical practice. Thus, when the 5% change in VC was used as an index, efficacy of the drug was evaluated with higher sensitivity than when the 10% change in VC was used. The 5% change in VC may seem only a slight change, but the annual decline in VC in the placebo group is said to be approximately 150 to 200 mL in many recent clinical trials [12,21-25]. In the phase III trial of pirfenidone, the annual decline in VC in the placebo group was 160 mL on average [12], and the mean baseline VC in the placebo group was 2472.3 mL, from which the annual rate of decline is calculated to be approximately 6.5%. That is, if a ≥ 10% change in VC is used as an index for evaluation over a period of a year, it may not be sensitive enough to detect efficacy of the drug, especially for changes within a shorter period of time such as 3 months and 6 months. Results of this sub analysis revealed that using a 5% change as an index improved the chances of detecting efficacy of the drug. Our results are considerably similar to those of extended analysis of the IFIGENIA study investigating the effect of N-acetylcysteine (NAC) in IPF, which also showed significance of a 5% threshold [26]. However, it should be noted that use of a smaller change as an index may require more accurate VC measurements.

According to the preceding report, the progression of disease was defined by the ≥ 10% decline in VC or death for evaluation of progression-free survival [12]. Results showed that the p-value of the difference between groups high-dose and placebo was 0.0280 and between groups low-dose and placebo was 0.0655. In this paper, the progression of disease was defined by the ≥ 5% decline in VC from baseline or death, and K-M plots were generated using thus defined PFS time. As a result, there were significant difference between groups high-dose and placebo and between groups low-dose and placebo (p = 0.0149 and p = 0.0034, respectively), (Figure 2-a) which seems to be more evident than those in the previous analysis by 10% decline [12]. When the progression of disease was defined by a ≥ 5% decline in VC from baseline on two successive occasions or death, the highly significant differences were also observed (Figure 2-b ), which supported the result of Figure 2-a.

Early identification of the response to therapeutic medication provides a clue in clinical decision making on treatment policy. We analyzed the positive/negative predictive values using the improvement ratings of months 3 and 12 based on 5% decline in VC. From the results of the differences of negative predictive values between placebo (50.8%) and pirfenidone (71.7%) groups, the efficacy of pirfenidone was also demonstrated (p = 0.0046). Thus, about 70% of patients assessed as non-progression at month 3 in pirfenidone group might remain in the state at 1 year. However, the results of the positive predictive values of placebo and pirfenidone groups showed that both values were very high, i.e., 86.1% and 87.1%, respectively. These results showed that the progression detected at month 3 remained (not reversed) at month 12 in most cases. These analyses suggested the possibility of identifying whether patients respond to pirfenidone or not at early phase after intervention, and of motivating patients to continue medication.

On the other hand, it will be a crucial question whether treatment should be withdrawn in patients who decline by ≥ 5% in VC at month 3. Patients with VC declined by 5% at month 3 generally had lower means of PaO2, VC, %VC, TLC, %TLC, and DLCO at baseline, and had higher means of SP-A, SP-D and dyspnea in daily living assessed with H-J classification score at baseline (Table 2). It was suggested that those patients with impairment of these baseline characteristics may lead to be corresponded to relatively "rapid progressors" in IPF, and treatment of any additional therapy would be recommended as soon as allowed. The effect of additional therapy strategies, such as combination with NAC [22] or BIBF-1120 [27], should be addressed in further clinical trials.

In order to translate the 5% decline in VC into a clinical relevant outcome, we compared the clinical outcomes (dyspnea in daily living assessed with H-J classification, mortality rate, and incidence of acute exacerbation) between 2 classes of patients, i.e., those with "worsened (VC ≥ 5% decrease)" and others with "non-worsened (VC < 5% decrease)" at month 3 (Table 3, 4). In short, dyspnea in daily living and mortality rate of patients with worsened at month 3 were significantly worsened after 1 year. Similar trend was also seen in the prevalence of acute exacerbation between the 2 classes of patients, which marginally supported the significance of the 5% change in VC. We speculated that the patients with 5% decline in VC at month 3 have further progression more easily; however, PFS times with origin at month 3 were not different between patients with or without 5% decline in VC at month 3 (Figure 3). Namely, it is noted that declines in VC at month 3 do not mean the possibility of further progression in next 9 months, i.e., month 3 to 12. In summary, except for the results of PFS times, it was suggested that a 5% decline in VC at month 3 is a clinically meaningful indicator in IPF and may be a useful prognostic factor. As the potential limitation, it should be addressed that these analytical results were obtained by the small number of subjects with death or prevalence of acute exacerbation within a one year study period.

## Conclusion

Results shown in this paper suggested that when 5% change in VC was used as an index instead of the 10% change, the efficacy of pirfenidone could be evaluated with higher sensitivity and robustness over the 12 month study. It was also shown by the results that the 5% change in VC at month 3 is suggested to be a clinically useful and significant promising prognostic factor of IPF.

## Abbreviations used in this paper

IPF: idiopathic pulmonary fibrosis; VC: vital capacity; FVC: forced vital capacity; TLC: total lung capacity; PaO_2_: alveolar-arterial oxygen tension; PFS: progression-free survival; SpO_2_: oxygen saturation by pulse oximetry; DLCO: diffusing capacity for carbon monoxide; FAS: full analysis set; PFT: pulmonary function test; 6MET: 6-minute steady-state exercise test; SP-A (or D): Surfactant protein-A (or D); K-M: Kaplan-Meier; BMI: Body Mass Index; H-J: Hugh-Jones; ATS: American Thoracic Society; ERS: European Respiratory Society.

## Competing interests

HT, ME, AA, YT, MS, HT, KN, AS, SK, and TN have received consultancy fees for advisary board, and HT, YK, ME, TO, AA, YS, and TN have received fees for speaking from Shionogi & Co., Ltd.

## Authors' contributions

HT and YK contributed equally to this extended analysis and should be considered co-first authors. All authors listed made significant conceptual and intellectual contributions to the design and conception of this phase III trial, substantially contributed to the article, and have provided final approval of the version submitted.

## References

[B1] King TE Jr Albera C Bradford WZ Costabel U Hormel P Lancaster L Noble PW Sahn SA Szwarcberg J Thomeer M Valeyre D du Bois RM INSPIRE Study Group Effect of interferon gamma-1b on survival in patients with idiopathic pulmonary fibrosis (INSPIRE.: A multicentre, randomized, placebo-controlled trial Lancet 2009 374 222 228 10.1016/S0140-6736(09)60551-1 19570573

[B2] du Bois RM Strategies for treating idiopathic pulmonary fibrosis Nat Rev Drug Discov 2010 9 129 140 10.1038/nrd2958 20094055

[B3] Raghu G Johnson WC Lockhart D Mageto Y Treatment of idiopathic pulmonary fibrosis with a new antifibrotic agent, pirfenidone: results of a prospective, open-label Phase II study Am J Respir Crit Care Med 1999 159 1061 1069 10194146 10.1164/ajrccm.159.4.9805017

[B4] Gahl WA Brantly M Troendle J Avila NA Padua A Montalvo C Cardona H Calis KA Gochuico B Effect of pirfenidone on the pulmonary fibrosis of Hermansky-Pudlak syndrome Mol Genet Metab 2002 76 234 242 10.1016/S1096-7192(02)00044-6 12126938

[B5] Nagai S Hamada K Shigematsu M Taniyama M Yamauchi S Izumi T Open-label compassionate use one year-treatment with pirfenidone to patients with chronic pulmonary fibrosis Intern Med 2002 41 1118 1123 10.2169/internalmedicine.41.1118 12521199

[B6] Azuma A Nukiwa T Tsuboi E Suga M Abe S Nakata K Taguchi Y Nagai S Itoh H Ohi M Sato A Kudoh S for the members of the Research Group for Diffuse Lung Diseases in Japan Raghu G Double-blind, placebo-controlled trial of pirfenidone in patients with idiopathic pulmonary fibrosis Am J Respir Crit Care Med 2005 171 1040 1047 10.1164/rccm.200404-571OC 15665326

[B7] Iyer SN Gurujeyalakshmi G Giri SN Effects of pirfenidone on transforming growth factor-beta gene expression at the transcriptional level in bleomycin hamster model of lung fibrosis J Pharmacol Exp Ther 1999 291 367 373 10490926

[B8] Gurujeyalakshmi G Hollinger MA Giri SN Pirfenidone inhibits PDGF isoforms in bleomycin hamster model of lung fibrosis at the translational level Am J Physiol 1999 276 L311 L318 9950894 10.1152/ajplung.1999.276.2.L311

[B9] Iyer SN Gurujeyalakshmi G Giri SN Effects of pirfenidone on procollagen gene expression at the transcriptional level in bleomycin hamster model of lung fibrosis J Pharmacol Exp Ther 1999 289 211 218 10087006

[B10] Misra HP Rabideau C Pirfenidone inhibits NADPH-dependent microsomal lipid peroxidation and scavenges hydroxyl radicals Mol Cell Biochem 2000 204 119 126 10.1023/A:1007023532508 10718632

[B11] Oku H Shimizu T Kawabata T Nagira M Hikita I Ueyama A Matsushima S Torii M Arimura A Antifibrotic action of pirfenidone and prednisolone: Different effects on pulmonary cytokines and growth factors in bleomycin-induced murine pulmonary fibrosis Eur J Pharmacol 2008 590 400 408 10.1016/j.ejphar.2008.06.046 18598692

[B12] Taniguchi H Ebina M Kondoh Y Ogura T Azuma A Suga M Taguchi Y Takahashi H Nakata K Sato A Takeuchi M Raghu G Kudoh S Nukiwa T Pirfenidone Clinical Study Group in Japan Pirfenidone in Idiopathic Pulmonary Fibrosis Eur Respir J 2010 35 821 829 10.1183/09031936.00005209 19996196

[B13] Latsi PI du Bois RM Nicholson AG Colby TV Bisirtzoglou D Nikolakopoulou A Veeraraghavan S Hansel DM Wells AU Fibrotic idiopathic interstitial pneumonia: The prognostic value of longitudinal functional trends Am J Respir Crit Care Med 2003 168 531 537 10.1164/rccm.200210-1245OC 12791580

[B14] Collard HR King TE Jr Bartelson BB Vourlekis JS Schwarz MI Brown KK Changes in clinical and physiologic variables predict survival in idiopathic pulmonary fibrosis Am J Respir Crit Care Med 2003 168 538 542 10.1164/rccm.200211-1311OC 12773325

[B15] Flaherty KR Mumford JA Murray S Kazerooni EA Gross BH Colby TV Travis WD Flint A Toews GB Lynch III JP Martinez FJ Prognostic implications of physiologic and radiographic changes in idiopathic interstitial pneumonia Am J Respir Crit Care Med 2003 168 543 548 10.1164/rccm.200209-1112OC 12773329

[B16] American Thoracic Society Idiopathic pulmonary fibrosis: diagnosis and treatment: international consensus statement: American Thoracic Society (ATS), and the European Respiratory Society (ERS) Am J Respir Crit Care Med 2000 161 646 664 10673212 10.1164/ajrccm.161.2.ats3-00

[B17] Zappala CJ Latsi PI Nicholson AG Colby TV Cramer D Renzoni EA Hansell DM du Bois RM Wells AU Marginal decline in forced vital capacity is associated with a poor outcome in idiopathic pulmonary fibrosis Eur Respir J 2010 35 830 835 10.1183/09031936.00155108 19840957

[B18] Clinical diagnostic and treatment guidance for idiopathic interstitial pneumonias Japanese Respiratory Society's Committee formulating diagnosis and treatment guideline for diffuse lung diseases 2004 Tokyo: Nankodo 63 65 [in Japanese]16679695

[B19] Nukiwa T Ebina M Takeuchi M Pirfenidone in idiopathic pulmonary fibrosis: From the authors Eur Respir J 2010 36 696 698 10.1183/09031936.00086910 19996196

[B20] Fletcher CM The clinical diagnosis of pulmonary emphysema; an experimental study Proc Royal Soc Med 1952 45 577 584 13003946

[B21] King TE Jr Safrin S Starko KM Brown KK Noble PW Raghu G Schwartz DA Analyses of efficacy end points in a controlled trial of interferon-gamma1b for idiopathic pulmonary fibrosis Chest 2005 127 171 177 10.1378/chest.127.1.171 15653980

[B22] Demedts M Behr J Buhl R Costabel U Dekhuijzen R Jansen HM MacNee W Thomeer M Wallaert B Laurent F Nicholson AG Verbeken EK Verschakelen J Flower CD Capron F Petruzzelli S De Vuyst P van den Bosch JM Rodriguez-Becerra E Corvasce G Lankhorst I Sardina M Montanari M High-Dose Acetylcysteine in Idiopathic Pulmonary Fibrosis N Engl J Med 2005 353 2229 42 10.1056/NEJMoa042976 16306520

[B23] Raghu G Brown KK Bradford WZ Starko K Noble PW Schwartz DA King TE Jr A Placebo-Controlled Trial of Interferon Gamma-1b in Patients with Idiopathic Pulmonary Fibrosis N Engl J Med 2004 350 125 33 10.1056/NEJMoa030511 14711911

[B24] Raghu G Brown KK Costabel U Cottin V du Bois RM Lasky JA Thomeer M Utz JP Khandker RK McDermott L Fatenejad S Treatment of idiopathic pulmonary fibrosis with etanercept: An exploratory, placebocontrolled trial Am J Respir Crit Care Med 2008 178 948 955 10.1164/rccm.200709-1446OC 18669816

[B25] King TE Jr Behr J Brown KK du Bois RM Lancaster L de Andrade JA Stähler G Leconte I Roux S Raghu G BUILD-1: a randomized placebo-controlled trial of bosentan in idiopathic pulmonary fibrosis Am J Respir Crit Care Med 2008 177 75 81 17901413 10.1164/rccm.200705-732OC

[B26] Behr J Demedts M Buhl R Costabel U Dekhuijzen RP Jansen HM MacNee W Thomeer M Wallaert B Laurent F Nicholson AG Verbeken EK Verschakelen J Flower CD Petruzzelli S De Vuyst P van den Bosch JM Rodriguez-Becerra E Lankhorst I Sardina M Boissard G IFIGENIA study group Lung function in idiopathic pulmonary fibrosis - extended analyses of the IFIGENIA trial Respir Res 2009 10 101 10.1186/1465-9921-10-101 19860915 PMC2774307

[B27] Selman M Pardo A Richeldi L Cerri S Emerging drugs for idiopathic pulmonary fibrosis Expert Opin Emerg Drug 2011 16 341 362 10.1517/14728214.2011.565049 21410428

